# Identification of effector-like proteins in *Trichoderma* spp. and role of a hydrophobin in the plant-fungus interaction and mycoparasitism

**DOI:** 10.1186/s12863-017-0481-y

**Published:** 2017-02-15

**Authors:** Paulina Guzmán-Guzmán, Mario Iván Alemán-Duarte, Luis Delaye, Alfredo Herrera-Estrella, Vianey Olmedo-Monfil

**Affiliations:** 1 División de Ciencias Naturales y Exactas, Departamento de Biología, Universidad de Guanajuato, Guanajuato, Gto, Mexico; 2 Laboratorio Nacional de Genómica para la Biodiversidad, Centro de Investigación y de Estudios Avanzados del IPN, Irapuato, Gto, Mexico; 3 Unidad Irapuato, Irapuato, Centro de Investigación y de Estudios Avanzados del IPN, Irapuato, Gto, Mexico

**Keywords:** *Trichoderma*, Effector, Plant-fungus interaction, Mycoparasitism, Hydrophobin

## Abstract

**Background:**

*Trichoderma* spp. can establish beneficial interactions with plants by promoting plant growth and defense systems, as well as, antagonizing fungal phytopathogens in mycoparasitic interactions. Such interactions depend on signal exchange between both participants and can be mediated by effector proteins that alter the host cell structure and function, allowing the establishment of the relationship. The main purpose of this work was to identify, using computational methods, candidates of effector proteins from *T. virens, T. atroviride* and *T. reesei*, validate the expression of some of the genes during a beneficial interaction and mycoparasitism and to define the biological function for one of them.

**Results:**

We defined a catalogue of putative effector proteins from *T. virens, T. atroviride* and *T. reesei*. We further validated the expression of 16 genes encoding putative effector proteins from *T. virens* and *T. atroviride* during the interaction with the plant *Arabidopsis thaliana,* and with two anastomosis groups of the phytopathogenic fungus *Rhizoctonia solani*. We found genes which transcript levels are modified in response to the presence of both plant fungi, as well as genes that respond only to either a plant or a fungal host. Further, we show that overexpression of the gene *tvhydii1,* a Class II hydrophobin family member, enhances the antagonistic activity of *T. virens* against *R. solani* AG2. Further, deletion of *tvhydii1* results in reduced colonization of plant roots, while its overexpression increases it*.*

**Conclusions:**

Our results show that *Trichoderma* is able to respond in different ways to the presence of a plant or a fungal host, and it can even distinguish between different strains of fungi of a given species. The putative effector proteins identified here may play roles in preventing perception of the fungus by its hosts, favoring host colonization or protecting it from the host’s defense response. Finally, the novel effector protein *TVHYDII1* plays a role in plant root colonization by *T, virens,* and participates in its antagonistic activity against *R. solani*.

**Electronic supplementary material:**

The online version of this article (doi:10.1186/s12863-017-0481-y) contains supplementary material, which is available to authorized users.

## Background

The genus *Trichoderma* includes soil borne filamentous fungi with a saprophytic life style [1]. Owing to the great capacity to secrete enzymes, shown by some species belonging to this genus, such as *T. reesei,* these fungi have been widely used in industry [2]. Additionally, species like *T. virens, T. atroviride, T. asperellum* and *T. harzianum,* are used as biocontrol agents because of their mycoparasitic behavior that allows them to antagonize major phytopathogenic fungi, such as *Botrytis cinerea* and *Rhizoctonia solani*, among others [3, 4].

In addition to plant protection by direct attack to pathogens, it has been shown that *Trichoderma* has the ability to establish beneficial interactions with plants (cotton, maize and *Arabidopsis thaliana)*, by promoting plant growth and development, possibly through the production of auxin-like compounds and other secondary metabolites [5–9], and enhancing the plant defense response against pathogens like *B. cinerea* and *Pseudomonas syringae,* promoting the accumulation of phytoalexins, salicylic and jasmonic acids [8, 10]. The molecular mechanisms that *Trichoderma* uses to establish beneficial interactions with plants are still largely unknown. However, effector-like proteins may play an important role in the establishment of the interaction, as occurs in the mycorhizal symbiosis or in the nitrogen fixing nodules. In these cases, proteins like MiSSP7 from *Laccaria bicolor* or NOD factors from *Rhizobium,* alter the physiology of the plant to promote the successful establishment of the biological relationship [11–13].

Effectors are secreted molecules that are capable of altering the plant cellular structure and/or function, allowing the pathogen to infect the plant [14–18], or the endophyte to colonize the roots [11, 13]. These effectors are classified in two main categories, according to their localization inside the plant: apoplastic effectors, acting from the intercellular space; and cytoplasmic effectors that can be translocated inside the plant cell [14, 15]. Many of the known cytoplasmic effectors have a specific translocation motif that would allow them to get inside the cell, such as the RXLR (arginine, any amino acid, leucine, arginine) motif of Avr3a from *Phytophthora* spp. [19], or a similar motif such as the RXLX motif of MiSSP7 [11]. Nevertheless, there is some controversy about the function of the RXLR motif. Some authors indicate that this motif is not sufficient or necessary for effector translocation [20]; although there are several reports that provide evidence for the relevance of this motif in various systems [19, 21–25]. Such is the case of the PfHRPII protein from *Plasmodium falciparum,* whose RXLX motif is sufficient to translocate the protein inside blood cells, or the RXLR motif of the Avr1B protein from *Phytophthora sojae* that allows translocation of the green fluorescent protein into soybean root cells [19, 26]. The latter suggests that this characteristic may be conserved among cytoplasmic effectors from different organisms.

In regard to effector functions in *Trichoderma*, the participation of some proteins in processes involved in plant defense has been demonstrated. Such is the case of Sm1 from *T. virens* and its ortholog Epl1 from *T. atroviride*, which are cerato-platanin proteins that induce reactive oxygen species accumulation and pathogen-related gene expression responses in maize and cotton [27, 28], and modulate resistance of tomato plants against *Alternaria solani, B. cinerea* and *P. syringae* [29]. Although, according to Gaderer and collaborators [30], Epl1 and Sm1 may not be the main proteins involved in the induction of plant defense responses, but rather Sm2 and Epl2. Similarly, TasSwo from *T. asperellum* induces the defense response system of cucumber against *B. cinerea* and *P. syringae,* promoting root colonization [31], and transcript levels of the hydrophobin Tashyd1 from *T. asperellum* increase during the interaction with cucumber plants [32]. In addition to the proteins mentioned above, some *Trichoderma* secondary metabolites such as harzianolide, 6-pentyl-α-pyrone and small antibiotic peptides known as peptaibols, can also induce the plant defense response [33, 34].

Effectors have been studied mainly in plant-pathogen systems, but their role in plant beneficial interactions is still poorly understood. Given the importance of effectors in the establishment of plant-microbe interactions, it is relevant to identify these molecules in order to study their functions and to gain a better understanding of the biological processes in which they participate. The availability of bioinformatic tools enables in silico analysis of whole genomes to search effector type molecules. These tools consider characteristics common to already identified and characterized effectors, such as the presence of a secretion signal peptide, short length-300 to 400 amino acids - cysteine rich regions, and presence of a putative translocation domain, among others [35–37].

There are some examples of possible effectors that have been identified using bioinformatic tools. Guyon and collaborators [37] identified 78 genes that codify for possible effectors within the secretome of the phytopathogen *Sclerotinia sclerotiorum*, considering features like phylogeny, gene selection and duplication, tridimensional protein prediction and possible function. The use of bioinformatic tools and *in silico* analyses have allowed researchers to distinguish effectors not only by their possible functions, but in some cases, these putative functions have been corroborated, like the effector Mg3LysM from the wheat pathogen *Mycospherella graminicola,* which was identified analyzing the genome of the fungus, and then its participation during the interaction with its host experimentally validated [38].

To our knowledge, there is very little evidence of effector-like proteins from filamentous fungi that could have a role in mycoparasitic interactions. Horner and collaborators [39] identified putative effectors from the mycoparasitic biocontrol agent *Pythium oligandrum* that were expressed during the interaction with the plant pathogen *Phytophthora infestans*; sequencing of cDNA libraries of *P. oligandrum* growing on *P. infestans* mycelium showed the expression of possible effectors, like crinkler effectors and elicitins, but no single RXLR-effector, suggesting that effectors could also participate in mycoparasitic interactions.

In this work, we focused on the identification of possible effector-like proteins among the predicted secretomes of *T. atroviride* and *T. virens* and the selection of some of the effector coding genes in order to validate their expression during the interaction with *A. thaliana* plants and in direct confrontation with the phytopathogen *Rhizoctonia solani*. We also evaluated the possible function of a Class II hydrophobin encoding gene in two biological interactions of *T. virens*, namely, root colonization and the antagonistic activity against phytopathogenic fungi.

## Results

- 1. Effector capacity of *Trichoderma* spp. decoded in extracellular proteins
  With the increased number of sequenced genomes, several computational algorithms to identify extracellular proteins have been developed, most of them, consider the existence of a signal peptide -SP- at the N-terminus that mediates secretion through the rough endoplasmic reticulum [40–43]. To select the most accurate available method to identify extracellular proteins, individual predictions, as well as, combinatorial analysis using the following algorithms: ProtComp-v9 [44], WolfPsort [45], SignalPv3-NN-D and SignalPv3-HMM-S [46], Phobius [47] and SignaLPv4-D [48] were performed (Additional file 1).
  All fungal protein sequences used to construct the evaluation datasets were retrieved from UniProtKB/Swiss-Prot, which is a repository of protein sequences manually curated. Using the query sentence described in the Additional file 1, we conformed 4 negative and 1 positive datasets (Additional file 2). These datasets were used to evaluate the ability of the above mentioned software to differentiate between extracellular proteins from non-extracellular proteins. Matthew’s correlation coefficient (MCC) was used to determine the best predictor. Table 1 shows that SignaLPv4-D was the most accurate predictor, closely followed by Phobius. We also found that the accuracy of using single programs is better than combining them (Additional file 3). According to this evaluation, SignaLPv4-D was used to predict classical extracellular proteins among the proteomes of *T. virens, T. atroviride* and *T. reesei*.

**Table 1:** System performance measures

Program	Positives		Negatives		Program performance evaluation			
	TP	FP	TN	FN	Rc	Pr	Acc	MCC
SignaLPv4-D^a^	74	2	398	4	0.949	0.995	0.987	0.954
SignalPv3-HMM-S/TMHMMv2	76	9	373	20	0.792	0.976	0.939	0.805
Phobius	74	8	392	4	0.949	0.980	0.975	0.910
ProtComp-v9	59	6	391	22	0.728	0.985	0.941	0.781
WolfPsort	56	7	392	23	0.709	0.982	0.937	0.759
SignalPv3-NN-D	76	34	366	2	0.974	0.915	0.925	0.781
SignalPv3-HMM-S	76	32	368	2	0.974	0.920	0.929	0.790
SignalPv3-NN-D/SignalPv3-HMM-S	75	15	370	18	0.806	0.961	0.931	0.777
WolfPsort/SignalPv3-NN-D	56	32	367	23	0.709	0.920	0.885	0.602
WolfPsort/SignalPv3-HMM-S	56	23	377	22	0.718	0.943	0.906	0.657
WolfPsort/Phobius	55	12	387	24	0.696	0.970	0.925	0.713

Performance was measured based on the program’s ability to correctly discriminate extracellular proteins from non-extracellular proteins

*Abbreviations*: *TP* true positive prediction, *FP* false positive prediction, *TN* true negative prediction, *FN* false negative prediction, *Rc* recall, *Pr* precision, *Acc* accuracy, *MCC* Mathew’s Correlation Coefficient

**Fig. 1:**
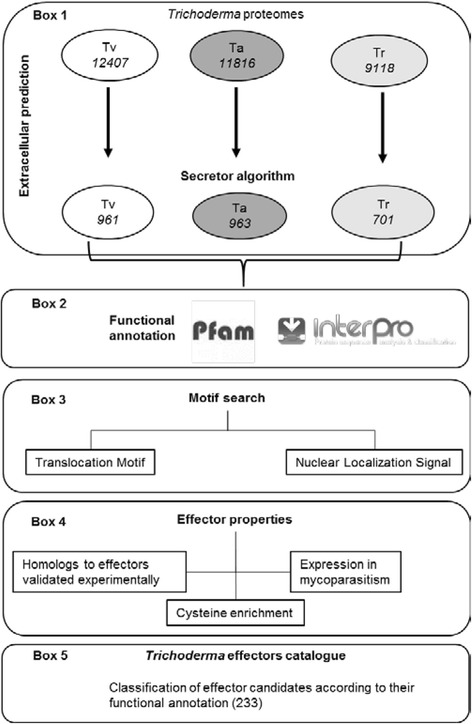
Bioinformatic pipeline for the identification of effector proteins in *Trichoderma* spp. The pipeline is composed of five major steps. Step 1 (*box 1*), extracellular proteins where predicted using the Secretor algorithm (designed and constructed for this work). Step 2 (*box 2*), functional annotation of the extracellular proteins, using Pfam database and InterPro pipelines. Step 3 (*box 3*) individual extracellular proteins where searched for translocation and nuclear localization signal motifs. Step 4 (*box 4*), individual extracellular proteins where annotated using current knowledge of effector properties. Step 5 (*box 5*), ranking and classification of the effector candidates based on the different effector properties matched by each protein. Tv, *Trichoderma* virens; Ta, *Trichoderma* atroviride; Tr, *Trichoderma* reseei
  A list of 233 putative effector-encoding genes were identified and grouped in 18 families according to their Pfam domains (Additional file 4). These domains were mainly related to activities like elicitor proteins, proteases, attachment to host surface and cell wall degrading enzymes. Although fewer, there were also proteins with domains related to mutualism through parasitism, signal transduction, response to stress and biosynthetic process. In addition, there were 12 proteins that showed no annotated Pfam domain. In total, there were 84 sequences from the proteome of *T. virens*, 86 from *T. atroviride* and 63 from *T. reesei*. Interestingly 102 of the 233 protein sequences have an RXLR or RXLR-like translocation domain (36 from *T. virens*, 38 from *T. atroviride* and 28 from *T. reesei*) that can be classified as putative cytoplasmic effectors.
  Belonging to the 233 effector candidates, 145 were grouped in 7 families known to participate in interactions with other organisms, and 31 out of these 145 coding sequences have an RXLR-like translocation domain. The 7 families mentioned above are: i) proteins with LysM repeats that can bind chitin fragments to prevent their perception by the plant, thus avoiding the activation of the plant immune responses [49]; ii) thioredoxin proteins that have been identified as possible effectors during plant-fungus [50] or mycoparasitic interactions [39] acting as antioxidants, facilitating the reduction of other proteins by cysteine thiol-disulfide exchange [51]; iii) proteins with CFEM (Common in Fungal Extracellular Membranes) domains, which have been linked as cell-surface receptors, signal transducers, or as adhesion molecules in host-pathogen interactions [52]; iv) cerato-platanin proteins that are able to elicit defense responses in plants like cotton or maize [27]; v) serine-proteases, implicated in fungal metabolism and appressoria formation [53]; vi) hydrophobins that are secreted proteins with high levels of hydrophobicity and eight conserved cysteine residues [54] located on the outer surfaces of cell walls of hyphae and conidia [55], where they mediate interactions between the fungus and the environment and have even been found to participate in the attachment of *Trichoderma* hyphae to the plant root [33] and appressoria formation in mycoparasitic interactions [56]; and vii) metalloproteases, known to be proteolytic enzymes from pathogenic fungi associated to tissue degradation [57].
  Although the list generated includes protein sequences from *T. reseei*, this research was focused on the sequences corresponding to *T. virens* and *T. atroviride* because they are well known to establish beneficial interactions with plants [5, 7–9] and their activity as mycoparasites has been widely confirmed [3, 4].
  To test if the proteins identified with Secretor could be involved in *Trichoderma-*host interactions, we selected 16 coding sequences from *T. virens* and *T. atroviride*, choosing at least one sequence from each of the 7 families of functions known to be involved in fungal interactions. Whenever possible, we chose those sequences containing an RXLR-like translocation motif (RXXX, XXLR, XXXR, XXLX, RXLX; Table 2), naming them according to their predicted function. The expression was determined in two types of interactions: a mycoparasitic and a beneficial *Trichoderma*-plant interaction.

^a^Most accurate method to identify extracellular proteins
  A script was generated -Secretor- to aid in the prediction of classical extracellular proteins of *T. virens, T. reesei* and *T. atroviride*. The script allowed us to search in the set of predicted extracellular proteins features known to be typical of experimentally validated effectors like the presence of translocation motifs among others (Fig. 1).

**Table 2:** Selected genes from *T. virens* and *T. atroviride* predicted effector encoding genes

Name	Species	JGI ID	Translocation Motif	Family
TvLysM1	*T. virens*	149422	RGYR	LysM Repeats
TvLysM2	*T. virens*	28703	RGYR	LysM Repeats
TvTrx1	*T. virens*	111061	HTYK	Thioredoxins
TvSep1	*T. virens*	230947	HALR	Serine-proteases
TvSep2	*T. virens*	11141	RRLR	Serine-proteases
TvSep3	*T. virens*	217176	RALR	Serine-proteases
TvHydII1	*T. virens*	49849	HGLR	Class II Hydrophobin
TvMp1	*T. virens*	86763	RLWR	Metalloproteases
TaLysM1	*T. atroviride*	291370	RGYR	LysM Repeats
TaTrx1	*T. atroviride*	161155	KNLK	Thioredoxins
TaTrx2	*T. atroviride*	139062	KELK	Thioredoxins
TaCFEM1	*T. atroviride*	302587	NA^a^	CFEM Domain
Epl2	*T. atroviride*	88590	RSLR	Cerato-platanin
TaSep1	*T. atroviride*	321810	HALR	Serine-proteases
TaSep2	*T. atroviride*	143106	RRLR	Serine-proteases
TaMp1	*T. atroviride*	314331	RLWR	Metalloproteases

^*a*^ *NA* not applicable
- 2. Expression of the selected genes during the *Trichoderma – A. thaliana* interaction
  To verify that the genes we selected were expressed during the beneficial *Trichoderma*-plant interaction we established interactions between *T. virens* or *T. atroviride* with *A. thaliana* seedlings, as previously reported [5] (Figs. 2a and 3a), and evaluated the expression of the selected genes at relevant time points of the interaction: when there was no physical contact between the organisms (BC), at initial contact of the mycelia with the plant root (C) and when the fungus overgrew the plant root (OG). The expression of the genes was determined by semi-quantitative RT-PCR, choosing 10 genes whose expression pattern was reproducible among four independent experiments (data not shown).

**Fig. 2:**
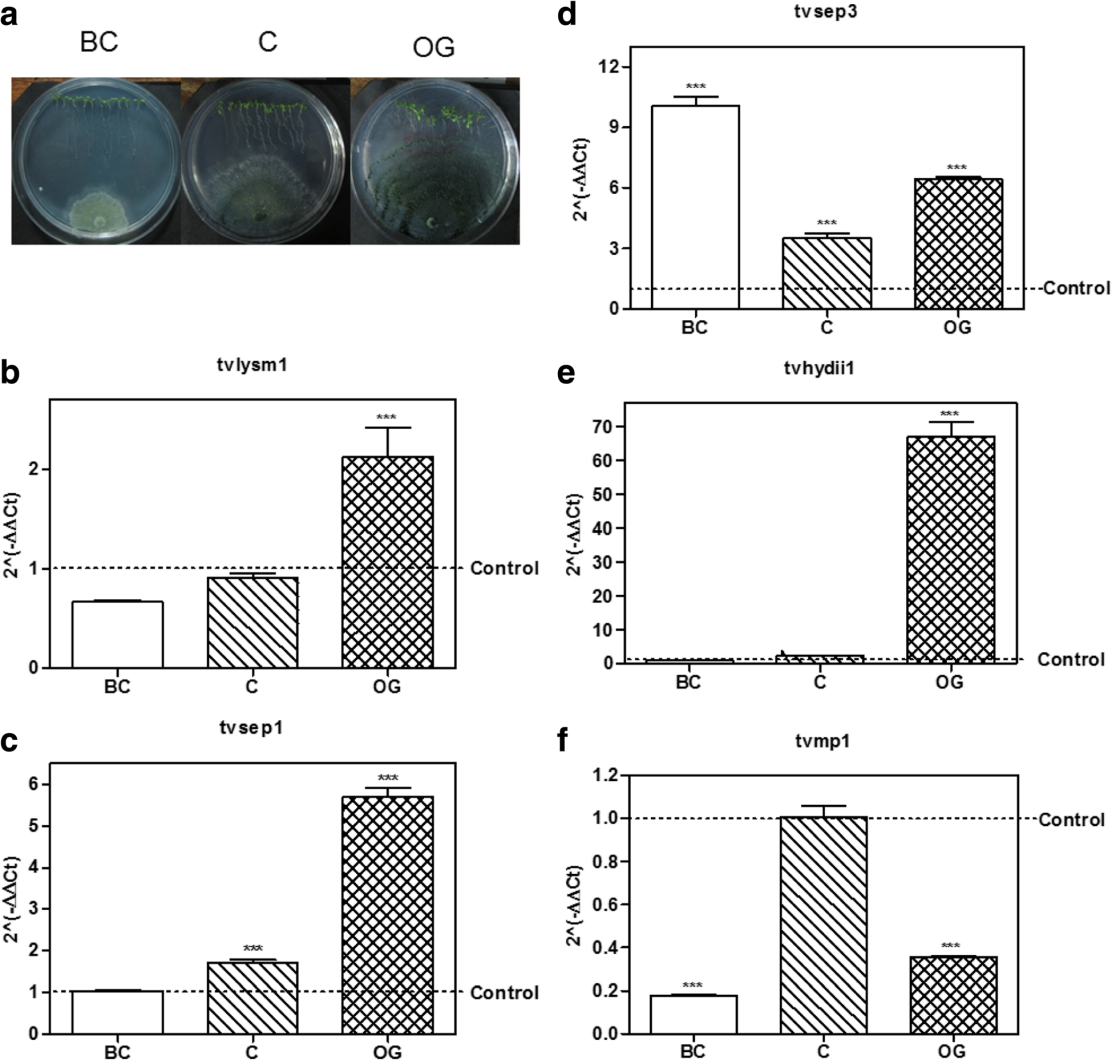
Expression of putative effector encoding genes from *T. virens* in interaction with *A. thaliana*. **a** *T. virens – A. thaliana* culture plates before contact (BC), at initial contact (C) and at overgrowth (OG) stages. **b**–**f**, expression level of the possible effector coding genes selected from *T. virens* at BC, C and OG stages of interaction with *Arabidopsis thaliana*. 2^(−ΔΔCt)^ values represent the change in the expression level of each gene compared to the control condition (*fungus growing alone*), which has a designated value of 1 (*dotted line*). Data were analyzed with a two-way ANOVA and a Bonferroni *post hoc* test; *** *p <* 0.001. Values represent means of three replicates, *bars* indicate standard deviation

**Fig. 3:**
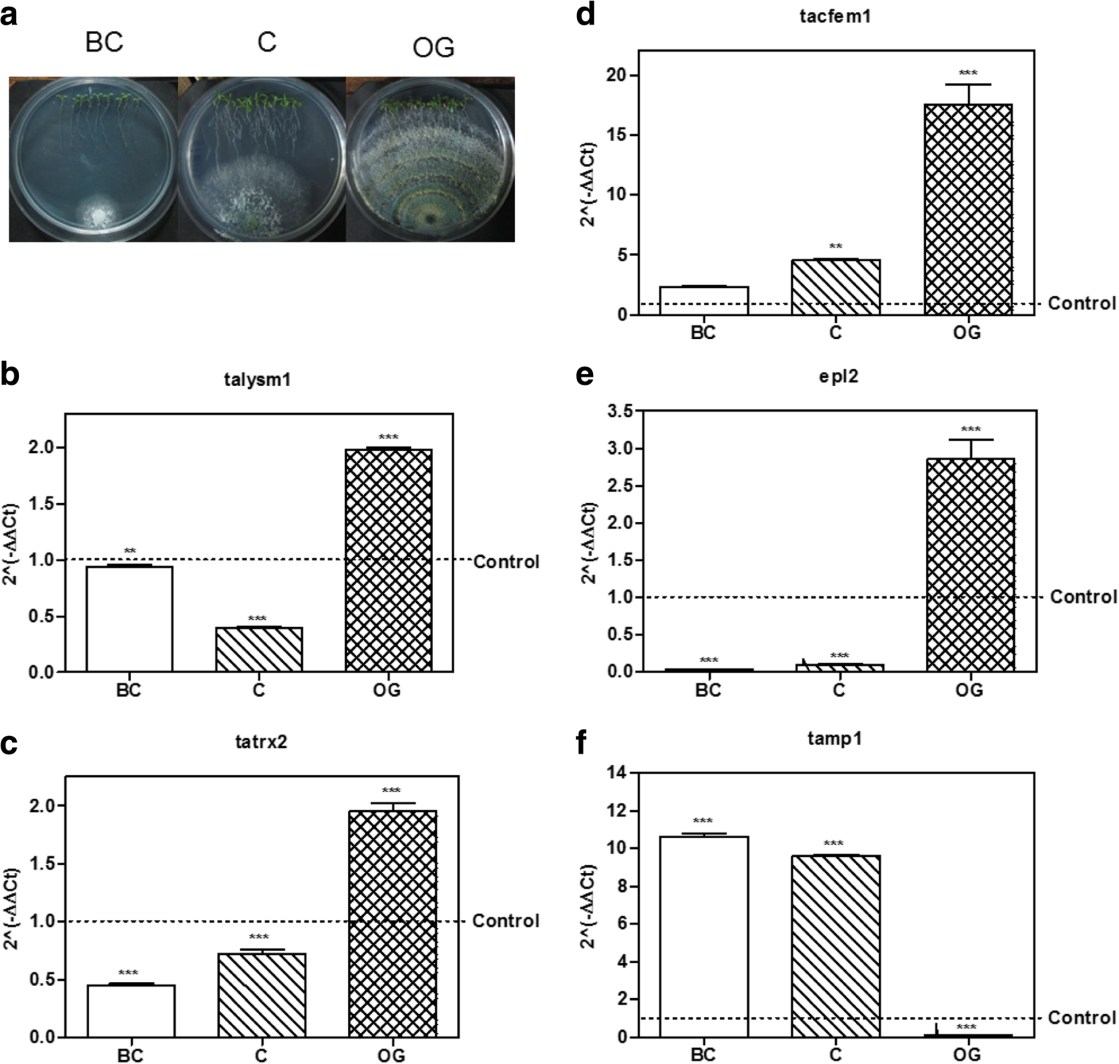
Expression of putative effector encoding genes from *T. atroviride* in interaction with *A. thaliana*. **a** *T. atroviride – A. thaliana* culture plates before contact (BC), at initial contact (C) and at overgrowth (OG) stages. **b**–**f**, expression level of the possible effector coding genes selected from *T. atroviride* at BC, C and OG stages of interaction with *Arabidopsis thaliana*. 2^(−ΔΔCt)^ values represent the change in the expression level of each gene compared to the control condition (*fungus growing alone*), which has a designated value of 1 (*dotted line*). Data were analyzed with a two-way ANOVA and a Bonferroni *post hoc* test; *** *p <* 0.001. Values represent means of three replicates, *bars* indicate standard deviation
  From the *Trichoderma-Arabidopsis* interaction assays, we used cDNA to validate by qRT-PCR the 10 sequences selected from the semi-quantitative RT-PCR results, and determine their level of expression at the three time points of interaction considered for this study (Figs. 2b–f and 3b–f).
  Regarding genes selected from *T. virens, tvlysm1*, a member of the LysM repeats family, showed 2.0-fold increase in expression as compared to the control mycelia, at the OG stage of the interaction (*p* < 0.001; Fig. 2b), while it remains unchanged at the BC and C stages (*p* > 0.05). The gene *tvsep1* that belongs to the serine-protease family was up-regulated (*p* < 0.001; Fig. 2c) at the C and OG stages of interaction with *A. thaliana,* with 1.7 and 5.7 higher expression than the control, respectively*,* and its expression didn’t change BC (*p* > 0.05). The gene *tvsep3* that also belongs to the serine-protease family, increased its expression at the three time points of the interaction (Fig. 2d), being its highest level of expression before contact with the plant (10.0 ± 0.78; *p <* 0.001). The gene *tvhydii1*, a member of the hydrophobin family, increased 66.6 ± 7.74 times its level of expression compared to the control when the fungus grew over the plant roots (*p* < 0.001; Fig. 2e), and remained unchanged during the first two stages of the interaction (*p* > 0.05). The gene *tvmp1* a putative metalloprotease, rather than increasing its expression level in the presence of the plant, was down-regulated (*p <* 0.001) at the BC and OG stages of the interaction (Fig. 2f).
  As for the genes selected from *T. atroviride*, the gene *talysm1* classified as a member of the LysM family was up-regulated (1.98 ± 0.03 increase) at the OG stage of the interaction (*p <* 0.001; Fig. 3b), while its expression remained unchanged before contact and it was down-regulated at the initial contact with the plant (*p <* 0.001). The gene *tatrx2*, a thioredoxin family member, increased its expression 1.95 ± 0.12 times compared to the control at the OG stage of the interaction (*p* < 0.001; Fig. 3c), while it was down-regulated at other stages (*p* < 0.001). Meanwhile the gene *tacfem1*, a member of the CFEM domain family, was up-regulated at C (4.58 ± 0.08 fold, *p* < 0.01) and OG (17.35 ± 2.93 fold, *p* < 0.001) stages of the interaction (Fig. 3d), and its expression showed no statistically significant differences before contact with the plant (*p* > 0.05). The gene with ID 88590 is annotated at the JGI database as *epl2,* a cerato-platanin family member, so we decided to use its annotated name instead. *Epl2* was up-regulated 2.83 ± 0.45 times compared to the control at the OG stage in co-culture with the plant (*p* < 0.001; Fig. 3e) and down-regulated during the first two stages of the interaction (*p* < 0.001). Only the gene *tamp1*, that was grouped in the metalloprotease family, was up-regulated at the BC and C stages of the interaction 10.64 ± 0.25 and 9.57 ± 0.14 times respectively (*p* < 0.001; Fig. 3f), but it was down-regulated at the OG stage (*p* < 0.001).
  These results show that the selected genes from *Trichoderma* spp. with the exception of *tamp1* and *tvmp1*, are mainly up-regulated at contact stages between *Trichoderma* and *A. thaliana* (i.e., at the C and OG stages of interaction). The expression patterns also suggest that these genes could participate in the establishment of the beneficial interaction due to their higher expression level in the presence of the plant.
- 4. Expression of the selected genes during the *Trichoderma-R. solani* interaction.
  The role of effectors in plant-fungus interaction is a research topic under strong investigation [14–16]. However, the role of effectors in interactions like mycoparasitism has not been significantly explored. Having in mind that *T. virens* and *T. atroviride* are well known effective mycoparasites against different major plant pathogens like *R. solani* [3, 4], and that the putative effectors could play a role in mycoparasitism, we decided to determine their expression levels during confrontation with two anastomosis groups of *R. solani*. We selected two anastomosis groups of *R. solani* because we have observed that in our laboratory conditions *Trichoderma* can limit in a more efficient way the growth of AG2, compared with that of AG5. This gave us the possibility to determine if some of the genes that we selected could have a host or even strain specific response, or if the observed increase in expression represents a general response to the presence of any potential host.
  We performed three independent confrontation assays between *T. virens* or *T. atroviride* with *R. solani* AG2 or AG5 (Fig. 4a). We collected *Trichoderma* mycelium before contact between the two fungi (BC); during initial contact (C); and at the time when *Trichoderma* overgrew *R. solani* (OG). By semi-quantitative RT-PCR, we selected the genes that had a reproducible expression pattern among the experiments (data not shown), and determined their expression level by qRT-PCR. Figure 4b–h shows the results from the qRT-PCR analysis of the interaction between *T. virens* or *T. atroviride* with *R. solani* AG5. The gene *tvlysm1* was up-regulated during the C and OG stages 1.8 ± 0.18 and 2.4 ± 0.17 times the expression in the control (*p <* 0.001; Fig. 4b). The gene *tvsep3* (Fig. 4c) increased its expression level during the contact and OG stages 12.8 ± 2.26 (*p <* 0.001) and 3.9 ± 1.87 (*p* < 0.05) times of that observed in the control, respectively. In contrast, the gene *tvhydii1* was up-regulated 3.7 ± 0.39 times the control only at the contact stage (*p* < 0.001; Fig. 4d). The gene *tvmp1* was up-regulated only at the first stage of the confrontation 1.7 ± 0.02-fold increase, relative to the control (*p <* 0.001; Fig. 4e), and it was down-regulated at the other stages (*p <* 0.001). These results show that, with the exception of *tvmp1*, the selected genes from *T. virens* could participate in the mycoparasitic interaction with *R. solani* AG5 mainly at the contact stage between both fungi.

**Fig. 4:**
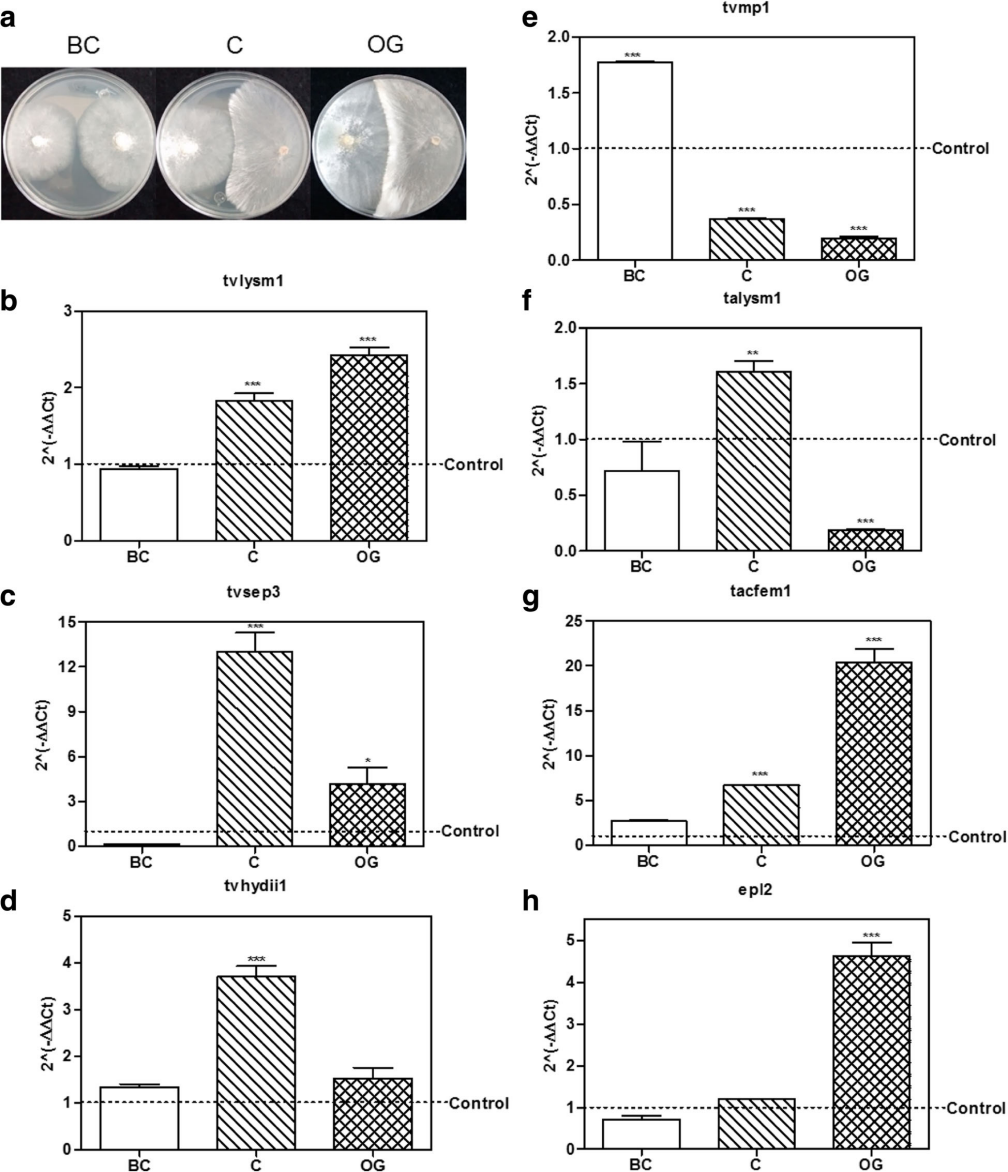
Expression level of putative effector encoding genes from *Trichoderma in* confrontation with *Rhizoctonia solani* AG5. **a** *T. atroviride – R. solani* AG5 culture plates before contact (BC), at initial contact (C) and at overgrowth (OG) stages. **b** *–* **e**, expression level of the selected genes *tvlysm1*, *tvsep3*, *tvhydii1* and *tvmp1* from *T. virens*. **f**-**g**, expression level of the selected genes *talysm1*, *tacfem1* and *epl2* from *T. atroviride*. BC, before contact between both fungi; C, initial contact of the fungi; OG, growth of *Trichoderma* over *R. solani* AG5. 2^(−ΔΔCt)^ values represent the change in the expression level of each gene compared to the control condition (*fungus growing alone*), which has a designated value of 1 (*dotted line*). Data were analyzed with a two-way ANOVA and a Bonferroni *post hoc* test; * *p* < 0.05; ** *p <* 0.01; *** *p <* 0.001. Values represent means of three replicates, *bars* indicate standard deviation
  In the case of the *T. atroviride* genes*, talysm1* increased its expression 1.6 ± 0.17 times relative to the control at the contact stage (*p <* 0.01; Fig. 4f), but it was down-regulated at the OG stage (*p <* 0.001). The expression of *tacfem1* was up-regulated at C and OG stages, 6.7 ± 0.02 and 20.2 ± 2.54 times respectively (*p <* 0.001; Fig. 4g). While *epl2* was up-regulated 4.6 ± 0.56-fold relative to the control until the OG stage (*p <* 0.001; Fig. 4h). These results indicate that the selected genes from *T. atroviride* may play a role in the mycoparasitic interaction with *R. solani* AG5 at stages where there is a direct contact between the two fungi.
  Figure 5a–d shows the results obtained for the selected genes from *T. virens* in confrontation with *R. solani* AG2. The gene *tvlysm1* was up-regulated in the C and OG stages, 1.6 ± 0.09 and 3.8 ± 0.43-fold increase, as compared to the control (*p* < 0.01, *p* < 0.001, respectively; Fig. 5a). However, this gene didn’t change its expression at the BC stage (*p* > 0.05), showing a similar pattern of expression in the interaction with AG5, where *tvlysm1* was also up-regulated at the C and OG stages (Fig. 4b). *tvsep3* was also upregulated 64.0 ± 5.6 and 62.3 ± 5.81 fold at the C and OG stages, relative to its expression in the control, respectively (*p <* 0.001; Fig. 5b); and was not significantly expressed at the BC stage (*p* > 0.05), showing also a similar pattern of expression in the confrontation with AG5 (Fig. 4c). The gene *tvhydii1* was up-regulated during the three stages of the mycoparasitic interaction with AG2 (*p <* 0.001; Fig. 5c), reaching its highest level of expression at the OG stage with 6.1 ± 0.98 fold, relative to the control. This gene showed a different pattern of expression in the interaction with AG5, where it was up-regulated only at the C stage (Fig. 4d). *tvmp1* was up-regulated only at the first stage (BC) of the confrontation 3.1 ± 0.28-fold increase (*p* < 0.001; Fig. 5d), showing a decrease in its expression level compared to the control at the OG stage (*p* < 0.001), as also observed in the interaction with AG5 (Fig. 4e). These results indicate that, with the exception of *tvmp1,* the selected genes from *T. virens* could participate in the mycoparasitic interaction with *R. solani* AG2 mainly in stages that involve direct contact between the two fungi.

**Fig. 5:**
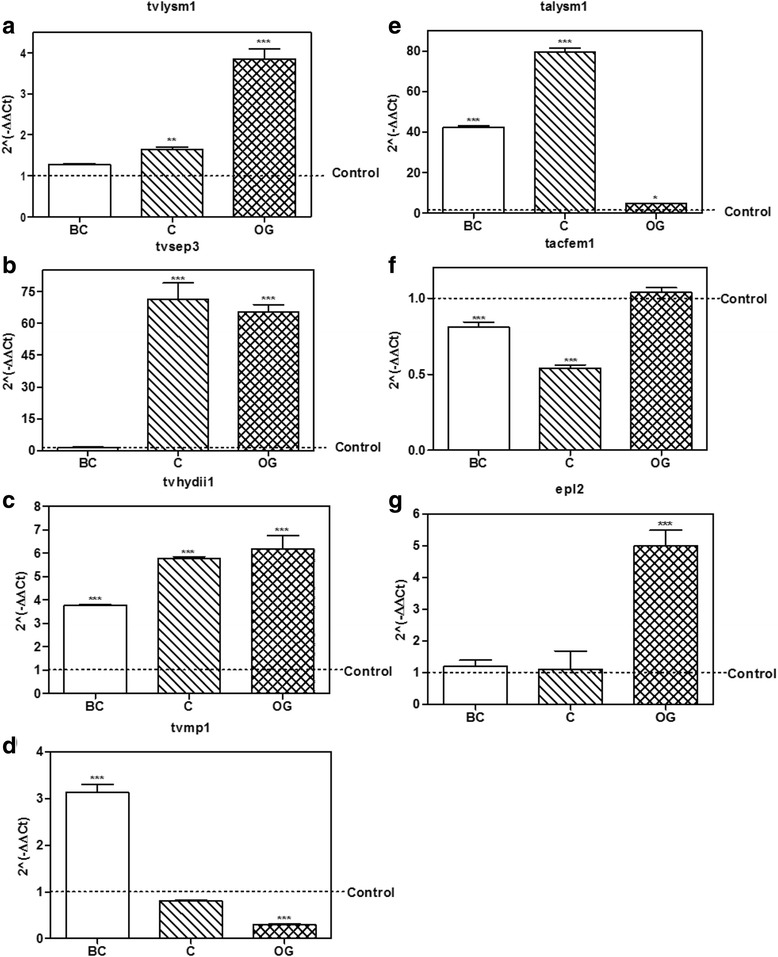
Expression level of putative effector encoding genes from *Trichoderma in* confrontation with *Rhizoctonia solani* AG2. **a** *–* **d**, expression level of the selected genes *tvlysm1*, *tvsep3*, *tvhydii1* and *tvmp1* from *T. virens*. **e**–**g**, expression level of the selected genes *talysm1*, *tacfem1* and *epl2* from *T. atroviride*. BC, before contact between both fungi; C, initial contact of the fungi; OG, growth of *Trichoderma* over *R. solani* AG2. 2^(−ΔΔCt)^ values represent the change in the expression level of each gene compared to the control condition (*fungus growing alone*), which has a designated value of 1 (*dotted line*). Data were analyzed with a two-way ANOVA and a Bonferroni *post hoc* test; * *p* < 0.05; ** *p <* 0.01; *** *p <* 0.001. Values represent means of three replicates, *bars* indicate standard deviation
  As for the genes selected from *T. atroviride*, *talysm1* was up-regulated at the three stages considered in the confrontation, with highest expression at the contact stage 79.4 ± 3.25 times, relative to the control (*p* < 0.001; Fig. 5e). This contrasts with its expression in the interaction with AG5, where E2 was up-regulated only at the contact stage (Fig. 4f). *tacfem1* was not up-regulated at any stage considered for the study. On the contrary, it was down-regulated at the BC and C stages (*p* > 0.05; Fig. 5f) showing a contrasting behavior to that observed in the interaction with AG5, where it was up-regulated at the C and OG stages (Fig. 4g). The gene *epl2* was up-regulated at the OG stage 4.9 ± 0.85 fold (*p <* 0.001; Fig. 5g). And at the other two stages of the interaction it didn’t change its expression, compared to the control (*p* > 0.05), showing a similar pattern of expression in the interaction with AG5 (Fig. 4h). These results indicate that *talysm1* and *epl2* could participate in the mycoparasitic interaction with AG2, but at different stages.
- 5. Knockout and overexpressing strains of *tvhydii1* gene from *T. virens.*
  Due to the expression pattern observed in both types of interaction, we selected the gene *tvhydii1,* encoding a class II hydrophobin family member from *T. virens*, to determine its possible role during the interaction with plants and/or mycoparasitism. We generated knockout and overexpressing strains for this gene. The null mutants *Δtvhydii1* T2.1 and *Δtvhydii1* T2.3 were selected after confirmation of the gene replacement event (Fig. 6a). The level of *tvhydii* transcript from the null mutants and the overexpressing strains *tvhydii*OE T2 and *tvhydii1*OE T5 were determined by RT-PCR, where mutants showed no presence of transcript, while OEs strains showed closed to 10-fold higher expression level than the expression observed in the wild type strain (Fig. 6b).

**Fig. 6:**
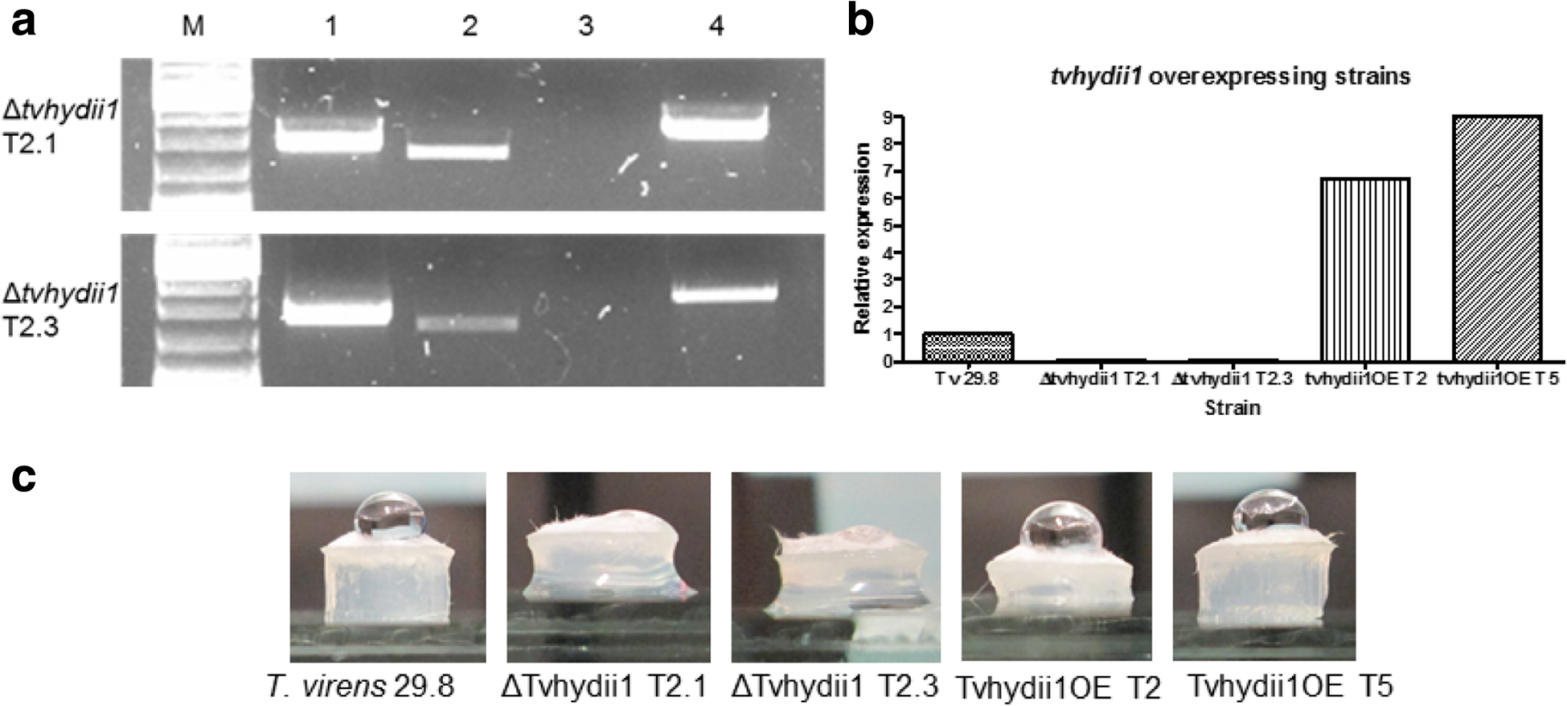
*tvhydii1* knockout and overexpressing strains. **a** confirmation of the *tvhydii1* gene deletion; *lane 1, 5’* end of the deletion cassette inserted at the *tvhydii1* locus; *lane 2*, *tvhydii1* wild type amplification as control; lane 3, *tvhydii1* amplification in the knockout strains; *lane 4, 3’* end of the deletion cassette inserted at the *tvhydii1* locus. **b** semi-quantitative RT-PCR of the knockout and overexpressing strains; RT-PCR reactions were carried out using *gpd* gene as amplification control for *tvhydii1* gene; **c** hydrophobicity assay, a 20 μL drop of distilled water was placed over plugs of 72 h mycelia of each *T. virens* strain
  Because *tvhydii1* encodes a class II hydrophobin, we decided to perform a hydrophobicity assay, placing a drop of distilled water over 72 h old mycellium plugs of the knockout and overexpressing strains [58] . As shown in Fig. 6c, while overexpressing strains are capable of mantaining the drop of water, the kockout strains are not. This result shows that knockout strains possibly do not have a hydrophobic surface capable of forming a drop of water, due to the absence of *tvhydii1*.
- 6. Role of *tvhydii1* in mycoparasitism.
  One of the functions of hydrophobins is attachment to different surfaces, like hyphae, participating in a mycoparasitic interaction [59, 60]. Our results of gene expression in the *T. virens-R, solani* mycoparasitic interaction suggested that *tvhydii1* could be involved, specially in the interaction with *R, solani* AG2. Therefore, we carried out confrontation assays between *R. solani* anastomosis groups AG2 and AG5 and the knockout and overexpressing strains.
  The confrontation plates between knockout and overexpressing strains against *R. solani* AG2 at 5 and 7dpi are shown in Fig. 7, when differences were more noticeable. At 5dpi, mycelium from both overexpressing strains is denser than the knockout strains, at the contact area with the pathogen. At 7dpi, overexpressing strains, specially strain T5, overgrow *R. solani* AG2, as evidenced by the size of the pathogen colony (Fig. 7 front plate) and the boundaries between strains (Fig. 7, back plate), in comparison to the knockout strains. We did not observed any significant difference in the interactions with *R. solani* AG5 (data not shown). This result shows that *tvhydii1* gene may be involved in mycoparasitic interactions, favouring growth of *T. virens* over the fungal host.

**Fig. 7:**
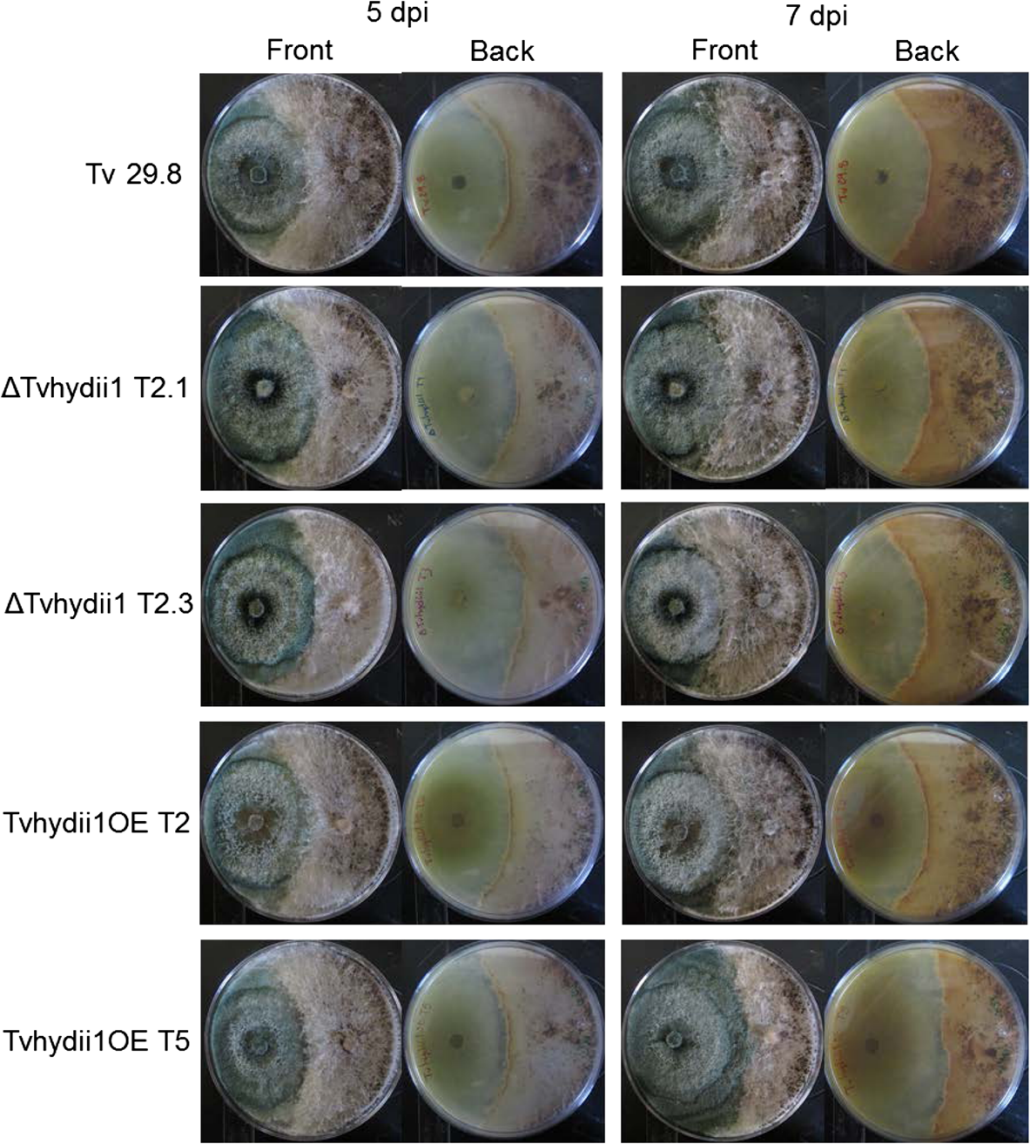
Mycoparasitic characterization of *tvhydii1* strains. Confrontation plates between *R. solani* AG2 and each *tvhydii1* strain at 5 and 7dpi. *Front*, photographs of the upper part of the plates; *Back*, photographs of the *bottom* part of the plates. Plugs of 72 h mycelia were placed in opposite parts of potato dextrose agar plates, confronting the selected *tvhydii1* strain with the phytopathogen. *Plates* were placed at 28 °C in total darkness for 14 days
- 7. Role of *tvhydii1* in root colonization.
*Trichoderma* also uses hydrophobins to attach to the surface of plant roots, facilitating root colonization [32, 56, 61]. Consequently, we used *Solanum lycopersicum* plants to determine the capacity of the strains to colonize plant roots. For this purpose, we extracted DNA from roots 3 weeks after inoculation with *T. virens*, and carried out qPCR of the *Trichoderma sm1* and the tomato *expressed* gene to establish the relative abundance of *T. virens* in plant roots [62].
  As shown in Fig. 8, we found *sm1* to be more abundant in the roots of plants inoculated with both overexpressing strains, than the plants inoculated with the wild type strain, suggesting that overexpression of *tvhydii1* results in more efficient colonization of plant roots. In agreement, *sm1* was less abundant in the roots of plants that interacted witn both knockout strains than in the plants inoculated with the wild type strain. Thus, our results indicate that *tvhydii1* plays a role in plant root colonization by *T. virens,* most likely facilitating attachment of hyphae to the plant roots.

**Fig. 8:**
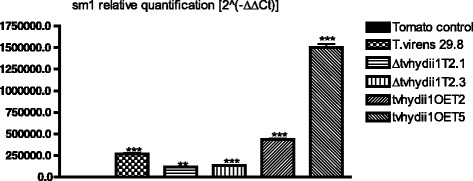
Root colonization assay of *tvhydii1* strains. Relative quantification of *sm1* amplification in tomato roots colonized by *T. virens* 29.8, Δ*tvhydii1* T2.1, Δ*tvhydii1* T2.3, *tvhydii1*OE T2 and *tvhydii1*OE T5. 2^(−ΔΔCt)^, values represent the amplification level of *sm1* gene relative to the amplification level of the *expressed* gene of the plant DNA, compared to the control condition (tomato DNA). *T. virens* 29.8 and *S. lycopersicum* DNA were used as amplification controls. Data were analyzed with a one-way ANOVA and a Bonferroni *post hoc* test; ** *p <* 0.01; *** *p <* 0.001. Values represent means of three replicates, *bars* indicate standard deviation

## Discussion

The identification of extracellular proteins from genome sequence data is not a new topic, so there are a lot of bioinformatic packages posted on the Web that allow you to solve this issue relatively easily. Thus, the issue is deciding which one is the best option for your data. In order to choose the best option, the most commonly used bioinformatic packages to identify extracellular proteins were evaluated. The evaluation showed that SignaLPv4-D was the best tool to predict extracellular proteins (Table 1). However, our main purpose was to identify effector proteins, since all effector proteins are extracellular proteins, but not all extracellular proteins are effectors, the prediction must distinguish the potential effectors coded in the extracellular dataset, so a pipeline (Secretor) was developed to predict a more accurate set of extracellular effector proteins.

While during the development of Secretor other two pipelines were published, the Fungal Secretome Database -FSD- [41] and the Fungal Secretome Knowledge Base -FunsecKB- [63]. Both pipelines eliminate ‘a priori’ sequences with: i) endoplasmic reticulum signal; ii) nuclear localization signal; iii) trans-membrane helix (es); iv) glycosylphosphatidylinositol -GPI anchors and*; v)* sub-cellular localization signals. However*,* it is widely known that many effectors contain signals that allow them to be transported to these sub-cellular compartments [22, 64–70]. The automated ‘Secretor’ pipeline filters extracellular proteins considering also putative extracellular proteins with sub-cellular localization signals different than extracellular. This is important to note because this set of potential effectors proteins is commonly discarded, making of this a strength of our algorithm.

Another consideration to make while searching for potential effectors are the characteristics that define them: homology to known effectors, secretion signal, sequence length, elicitor function, conserved motifs, etc. Previous efforts have led to the identification of putative effector proteins among *Trichoderma* species, as reviewed by Horwitz and collaborators [71], where it is mentioned that taking into consideration only one or two of these characteristics to search for potential effector proteins, may be misleading because some of them should then be discarded, especially when homology is the main criterion used. Some of the effectors annotated for *Trichoderma* spp. that work includes cerato-platanin proteins such as Sm1/Epl1, Swollenin, hydrophobins, LysM effectors and some homologues to known effectors in *Magnaporthe* spp. and *Laccaria bicolor*.

To identify and classify candidate effectors of the three *Trichoderma* genomes, we used current knowledge of the properties mined from experimentally validated fungal effectors (Fig. 1). The pipeline identifies 233 effector candidates encoded in the genomes of the three *Trichoderma* species that passed the established criteria. The set of effector candidates includes: elicitors, hyphae protectors, proteins involved in pathogenesis, transcriptional regulators, signal transducing proteins, catabolic enzymes and a set of proteins without functional domain and/or annotation.

One important work that searched for potential effectors is that of Schmoll and collaborators [72]. They identified a set of over 2500 putative small secreted proteins (SSPs) among the genomes of *T. virens, T. atroviride* and *T. reesei*, focusing primarily in known effector characteristics such as, the presence of a secretion signal and the length of the predicted sequence (300 amino acids or less). Our set of potential effectors includes not only SSPs, but also a wider range of proteins that could be effectors. All of the sequences that we identified (100%) among the cerato-platanin, class II hydrophobins, cellulases and glycoside hydrolases families were also identified by Schmoll and collaborators [72]. Regarding other main effector families in our catalogue, 84.4% of the CFEM proteins and 58.8% of the LysM proteins that we identified were found in the study mentioned above. Other functional categories were shared between both lists in a lower proportion, such as thioredoxins (27.3%), metalloproteases (16.7%) and serine proteases (2.1%). But we also identified other potential effector families such as aspartil proteases, FAD-binding proteins, and oxidoreductases, among others, which means that our study covers all types of SSPs identified by them and includes other possible candidates.

In this study, we determine that the coding sequences we selected from our *Trichoderma* catalogue of possible effector coding genes are transcriptionally active during interactions with plant and fungi. Lamdan and collaborators [73] obtained 280 secreted proteins from the interaction of *T. virens* with maize roots in a hydroponic system, including Sm1 and several proteins with CFEM domain. We predicted 84 possible effector proteins from *T. virens,* and 22 out of the 84 effector coding sequences are represented among their secretome, belonging to different families of predicted functions: five small secreted cysteine rich proteins (SSCPs) one LysM protein, one glycoside hydrolase, six aspartyl proteases, eight serine proteases and one metalloprotease (TvMp1).

It is important to note that, as mentioned above, our algorithm was designed to predict potential effector proteins considering that not all secreted proteins are effectors. From the 963 sequences classified as secreted proteins from the *T. virens* genome, our algorithm predicts that 8.7% corresponds to effector proteins. The 22 proteins that are shared between our catalogue and the secretome described by Lamdan and collaborators [73], represent a very similar value (7.8%) to the percentage that we determined, which could correspond to effectors.

Considering the possibility that selected genes among our *Trichoderma* effector catalogue could participate in the establishment of the *Trichoderma-A. thaliana* interaction and in mycoparasitism, our results allowed us to identify those genes that: a) respond to the plant and the fungi; b) only to the fungi; or c) to the plant and just one of the fungal anastomosis groups tested. The genes that were up-regulated in the presence of the plant and the fungi are *tvlysm1*, *tvsep3* and *tvhydii1* from *T. virens*, and *talysm1* and *epl2* from *T. atroviride*, suggesting that these genes may respond in general to the presence of any potential host. The gene *tvmp1* was the one that responded only to the presence of both anastomosis groups of *R. solani*, and not to the presence of *A. thaliana,* so this gene could play a role particularly in mycoparasitic interactions of *T. atroviride.* The gene *tacfem1* responded to the plant and only to *R. solani* AG5 but not *R. solani* AG2, indicating that this gene could respond to plant hosts and could be specific to a fungal anastomosis group.

Regarding each gene family and its possible function, the members of the LysM repeats family *tvlysm1* and *talysm1*, which are orthologs with 96% identity*,* increase their expression during direct physical contact between *Trichoderma* and *A. thaliana* or *R. solani*. It is known that this kind of proteins bind chitin oligomers from the fungus, with the consequence that plant LysM receptors are avoided and plant defense responses are not triggered [74–76]. Examples are the effector Mg3LysM from *Mycosphaerella graminicola* [38] or Ecp6 from *Cladosporium fulvum*, which also binds chitin fragments [77]. In addition, LysM proteins can protect fungal hypha from plant hydrolytic enzymes like Mg3LysM [38] and possibly from hydrolytic enzymes from fungal preys in a mycoparasitic interaction. This suggests that *tvlysm1* has a role preventing *Trichoderma* detection by its plant host, and *talysm1* protect *Trichoderma* from its fungal prey.

Expression of the member of the thioredoxin family *tatrx2*, was evaluated in the *Trichoderma*-plant interaction. It was found to be up-regulated only when there was contact with the plant. Recent studies have suggested that thioredoxins may play an important role as effectors, due to their ability to bind directly to the apoptosis signal-regulating kinase 1 -ASK1-, thus inhibiting the activation of stress-activated MAPK signaling cascades that lead to apoptosis [78]⁠. Thioredoxins have been reported as putative effector proteins in *U. maydis* [50]*, L. bicolor* [79], and the mycoparasite *Pythium oligandrum* [39]*,* although their true role in pathogenesis remain uncovered yet. Also, Alkhalfiuoi and collaborators [80] found two thioredoxins from *Medicago truncatula,* Trx s1 and Trx s2, which were up-regulated during symbiosis with *Sinorhizobium meliloti,* suggesting that these proteins may be involved in the establishment of the symbiosis. The product of the gene *tatrx2* could have a role in mediating the beneficial interaction between *Arabidopsis* and *T. atroviride,* and possibly inducing plant resistance.

The member of the CFEM family, *tacfem1*, is not up-regulated in the presence of *R. solani* AG2, but is induced in the interaction with *R. solani* AG5 at the OG stage. CFEM proteins have functions related to signal transduction and adhesion to surface in host-pathogen interactions [52]. Some CFEM proteins are involved in appressoria formation, like Pth11 from *M. griseae* [81] playing a role in colonization of the host, which suggests *that tacfem1* may have a role in host colonization of plant and fungi. In agreement with our results, Reithner and collaborators [82] reported induction of this gene in confrontation assays with *R. solani* AG-4 and *P. capsici*. The possible role of these effectors in *Trichoderma* needs further investigation, however, the fact that at least one of them is expressed during the *Trichoderma-*host interaction suggests a general response involved in the crosstalk communication with other organisms.

The gene *epl2,* a member of the cerato-platanin family, increases its expression at the contact stages with both plant and fungi. Recently Gaderer and collaborators [30] demonstrated that Epl2 is involved in the protection of maize against *Cochliobolus heterostrophus* and may have a more important role than Epl1. Thus, it makes sense that *epl2* is upregulated during the interaction with the plant in our experimental conditions. As far as we know, there is no report of the possible role of this gene in mycoparasitic interactions of *T. atroviride*. Our results show that *epl2* increases its expression in the presence of *R. solani* when *T. atroviride* is growing over it, suggesting its possible role in mycoparasitism. Further investigation is required, however, to determine whether or not it is involved in the *Trichoderma*–fungus interaction.

Regarding the members of the serine-proteases family, the gene *tvsep1* was evaluated only in interaction with the plant, where it was upregulated just before contact and in contact with the plant. And the gene *tvsep3* was upregulated during the interaction with the plant, and at the contact stages with both fungi. Due to the hydrolytic activity of serine-proteases in degrading peptide bonds, *tvsep1* and *tvsep3* could be involved in protecting the *Trichoderma* hyphae from host catalytic enzymes. Pozo and collaborators [83] showed that cotton seedlings treated with strains of *T.* virens that overexpress the gene that codifies for a serine-protease, *tvsp1* showed less disease symptoms when infected with *R. solani*. This gene was upregulated in the presence of different fungal cell walls. So, it would be interesting to investigate if these serine-proteases play a similar role in the *Trichoderma-*plant interaction.

The member of the class II hydrophobin family tested in this study was the gene *tvhydii1* that was upregulated at contact stages with the plant, and both *R. solani* AG5 and AG2. These types of proteins are involved in surface attachment of the fungus to its host. In plant-fungus interactions, they play a role in root colonization, like the class I hydrophobin Tashyd1 of *T. asperellum* [32] or MHP1 from *M. griseae* that increases its expression during rice leaf colonization [56]. Hydrophobins can play an important role in plant-fungus interactions, such as HYTLO1 from *T. longibrachiatum,* that induces plant defense related genes, and displays antifungal activity against phytopathogens [84], indicating their possible role as effectors. We found that knockout strains from *tvhydii1* have a reduced capacity to colonize tomato roots, and overexpression of this hydrophobin encoding gene enhances root colonization by *T. virens.* In reference to the role of hydrophobins in mycoparasitism, two hydrophobin genes from *Phlebiopsis gigantea, Pgh1* and *Pgh2,* are highly expressed during confrontation with the pathogen *Heterobasidium annosum*, suggesting their role in mycoparasitism [59]. We found that the overexpression of *tvhydii1* restricts colony size of *R. solani* AG2, compared to the wild type strain, but there was no difference with *R. solani* AG5. The hydrophobin encoding gene *HFB2-6* from *T. asperellum* is upregulated in the presence of *A. alternata* cell walls and poplar root and stem powder, suggesting the role of this hydrophobin in interaction with both plant and fungal hosts [85], which may be a similar situation for *tvhydii1*, that is expressed in the presence of *Arabidopsis* and *R. solani*, and could be involved in attachment to fungal hyphae and plant roots, according to our results.

As for members of the metalloproteases family, the gene *tvmp1* is upregulated only before contact occurred between the two fungi. But not at the contact and overgrowing stages, and it is not expressed in the presence of *A. thaliana*, indicating that this gene may be involved during the early stages of these interactions. The gene *tamp1* was evaluated only in interaction with *A. thaliana* where it is upregulated when there was no contact between the plant and *T. atroviride.* Suggesting that it may participate at early stages of the interaction. The protein Avr-Pita from *M. grisaea,* which has homology to metalloproteases from *Aspergillus* spp. and *Penicillium*, induces resistance in rice [86, 87]. In analogy, it would seem possible that *tamp1* plays a role at inducing plant resistance too.

## Conclusion

The establishment of successful microorganism’s interactions depends on a signal exchange between participants. This dialogue involves many molecules that can be specific to particular interactions. Here we provide evidence that some of the genes respond in a general manner, either to plant or fungal hosts, while other genes appear to respond with some specificity towards plants or fungi or to specific strains, indicating that the fungus may be able to respond in different manners according to its host. We confirmed that, at least one of the genes that we identified as possible effector coding gene, could be involved in both mycoparasitism by *T. virens* and the plant beneficial interaction. Our data represents an important step towards the identification of genes that respond during the *Trichoderma*-plant interaction and mycoparasitism.

## Methods

### Selection of possible effector coding genes from *Trichoderma* spp.

To identify possible effector proteins among the secretomes of *Trichoderma* spp. we first evaluated the prediction accuracy of several computational algorithms to identify extracellular proteins, evaluating both individual and combinatorial predictions of the following algorithms: ProtComp-v9, WolfPsort, SignalPv3-NN-D, SignalPv3-HMM-S, Phobius and SignaLPv4-D. For this, we used genome datasets from predicted extracellular proteins of ascomycete fungi [63, 88], predicted proteome datasets for *T. virens, T. atroviride* and *T. reesei* from the JGI website [89] and the National Center for Biotechnology Information [90] and a Pubmed search for experimentally validated effector proteins from fungi [91]. We obtained four negative and one positive dataset to perform the evaluation and the programs were run locally with the latest free available distribution. In order to assess each prediction program performance, several statistical values were calculated. In the following formulas we indicate with TP and TN the number of True Positive and True Negative predictions, respectively, and with FP and FN the number of False Positive and False Negative predictions, respectively.

The recall (Rc), or true positive rate, was calculated as the number of proteins correctly predicted as extracellular over the total number of positive examples. Rc = TP /TP + FN. The precision -Pr-value corresponds to the number of proteins correctly predicted as extracellular over the total number of protein predicted as extracellular. Pr = TP /TP + FP. The false positive rate (Fpr) corresponds to the number of protein predicted as extracellular but annotated as negative. Fpr:FP/FP + TN. The accuracy (Acc) is the proportion of all true predictions on positive and negative examples and is given by: Acc:TP + TN/TP + TN + FN + FP.

Finally, MCC is given by: MCC = (TP*TN)-TN*FN)/√(TP + FN) (TP + FP) (TN + FP) (TN + FN). It varies between −1 and +1; a value of +1 indicating there is a perfect agreement between observations and predictions; a value of 0 indicating that predictions are not better as they were randomly generated; a value of −1 indicating that predictions are in total disagreement with what is observed.

To identify possible extracellular proteins in *Trichoderma* species at genomic level, we designed the algorithm *Secretor*, which allowed us the prediction of *Trichoderma* extracellular proteins with a secretion signal peptide and no transmembrane domains. Functional characterization of extracellular proteins in *Trichoderma* was based on GO annotation and carried out by the platform Blast2GO [92]. Then, we searched for effectors among the extracellular proteins from *Trichoderma* spp. by using the proteins in the positive effector database as BLAST queries. Among significant hits (e-value < 0.001) we look for a series of characteristic features of effectors. These included known Pfam domains present in effectors; translocation motifs validated experimentally as RxLR [19], RxFLAK [64], W/Y/F-XC [23] and possible variants [RKH]x[LYMFYW][RKH] [93] between amino acids 15 to 75; nuclear localization signal (NLS); *de novo* motifs in extracellular proteins; tandem repeats and small secreted cysteine rich proteins (SSCRP). Finally, we ranked and clustered the predicted proteins according to the effector properties identified from the complete sets of *Trichoderma* proteomes. For complete details about the evaluation of the different programs for extracellular protein prediction and the design of the Secretor algorithm, see Additional files 1, 2 and 3. Out off the list of possible effector coding genes from *T. virens* and *T. atroviride* we selected 16 sequences, considering as main criteria that those sequences were representative for the seven main families in which the genes were classified and that they had preferentially an RXLR or RXLR-like translocation motif.

### *A. thaliana-Trichoderma* interactions assays

Seeds of *A. thaliana* Col-0 were surface sterilized with ethanol four times and then placed in 0.2X Murashige-Skoog agar plates, 10 seeds per plate, and incubated at 24 °C in a plant growth chamber (MRClab, model PGI-500H) with 16/8 h light/dark cycles. *T. virens* Gv29- 8 and *T. atroviride* IMI 206040 inoculation was carried out 4 days after germination of the seeds [5]. Plates were incubated for 3, 5 and 7 days, corresponding to before contact (BC), contact (C) and overgrowth (OG) stages tested. At each time, mycelia from the culture edge (1 cm in width) were collected for total RNA extraction and further cDNA synthesis. The corresponding *Trichoderma* strain was grown alone as control. Four independent experiments were performed.

### *R. solani-Trichoderma* interaction assays

For mycoparasitism confrontation assays, *T. virens* Gv29- 8 and *T. atroviride* IMI 206040 were grown on potato dextrose agar plates, covered with cellophane and grown at 28 °C in total darkness. Both *T. atroviride* and *T. virens* were confronted with *R. solani* AG5 and AG2 or with the same *Trichoderma* species as control. The mycelia from *Trichoderma* were collected at three time points: 0.5 cm before contact occurred between the two fungi (BC); during contact of both strains (C) and 0.5 cm after *Trichoderma* had overgrown *R. solani* (OG). Three independent experiments for each confrontation were performed.

### RNA extraction and cDNA synthesis

The collected mycelia from both types of interactions were frozen immediately in liquid nitrogen. Mycelia were ground to a fine powder under liquid nitrogen and total RNA was isolated using the TRizol method. cDNA was synthesized with RevertAid H Minus First Strand cDNA Synthesis Kit® (Thermo Scientific), following the manufacturer’s recommendations.

### Gene expression analysis by semi-quantitative RT-PCR

RT-PCR reactions were carried out with 50 ng of cDNA as template and 25 amplification cycles. The glyceraldehyde-3-phosphatedehydrogenase gene (*gpd*), a constitutive gene [94], was used as control of expression, and amplification of a fragment from the *sm1* gene from *T. virens* and *epl1* gene from *T. atroviride* were used as a positive control, based on its increased expression reported in the presence of plants [27, 28]. The densitometry analysis of the semi-quantitative RT-PCR assays was made using the ImageJ 1.48 V software (Wayne Rasband, National Institute of Health, USA), using the expression intensity from *gpd* to normalize the data.

### Gene expression analysis by qRT-PCR

qRT-PCR reactions were performed with Fast SYBR Green Master Mix® (Applied Biosystems) using 20 ng of cDNA as template. The glyceraldehyde-3-phosphatedehydrogenase gene (*gpd*) was used as housekeeping gene and three technical replicates were analyzed for each type of interaction. The ΔΔCt method was used to analyze the data obtain from the qRT-PCR assays, using the StepOne software (Applied Biosystems) to determine the expression of each selected gene.

### *Tvhydii1* knockout strains

To generate knockout mutants of *tvhydii1* gene from *T. virens*, we follow the Double Joint PCR protocol [95]. We performed 3 rounds of PCR to generate an interruption cassette containing the gene *arg2* (carbamoyl phosphate synthase, which reverts the arginine auxotrophy in the *T. virens* 10.4 strain, as reported by Baek & Kenerley [96], flanked by the upstream and downstream regions of the *tvhydii1* gene. These PCRs were performed with High Fidelity PCR Enzyme Mix (Thermo Scientific ®) as follow: PCR1, we amplified separately, the *tvhydii1* upstream and downstream regions (1500 bp each) and the ORF of wild type *arg2* gene, using the specified primers in Additional file 5; PCR2, this reaction takes place using the purified fragments from PCR1, to join the three fragments into the interruption cassette; PCR3, using nest primers, we amplified the fragment from PCR2 to obtain a sufficient amount of the interruption cassette for protoplast transformation. We followed the protocol reported by Castellanos and collaborators [97] to obtain and transform *T. virens* protoplasts, using 20 μg of purified PCR3 product. Transformants were verified using primers flanking the interruption cassette (see Additional file 5), to make sure the deletion was successful, and two of them were chosen for single spore selection. After four single spore rounds, no wild type gene amplification was detected, indicating that the deletion of the gene was successful. Knockout strains were maintained all the time in Vogel minimal medium.

### *Tvhydii1* overexpressing strains

To overexpressed the *tvhydii1* gene, we used the expression vector pUE08 [98] which has the selection marker *hph* (hygromycin phosphotransferase, that confers resistance to the antibiotic hygromycin) under the constitutive promoter of the pyruvate kinase gene (*pki*) of *T. reesei*. We amplified the wild type *tvhydii1* ORF by PCR using the primers Tvhydii1OE-F and Tvhydii1OE-R (Additional file 5) and then it was cloned into pUE08, using *Bam*HI and *Hind*III restriction sites. Overexpression was verified in the transformants, after four single spore rounds by RT-PCR, using the primers Tvhydii1 S1 and Tvhydii1 AS (Additional file 5). We extracted total RNA from mycelium cultivated during 48 h and cDNA was synthetized. RT-PCR reactions were carried out with 50 ng of cDNA as template, and 25 amplification cycles. The glyceraldehyde-3-phosphatedehydrogenase gene (*gpd*) [94], was used as control of expression. Overexpressing strains were maintained all the time in potato dextrose medium supplemented with 50 μg/mL of hygromycin.

### Hydrophobicity assay

We performed a hydrophobicity assay of the overexpressing strains and the knockout strains [58]. Over mycelium plugs of 72 h cultures from *T. virens* wild type strain, Δ*tvhydii1* T2.1, Δ*tvhydii1* T2.3, *tvhydii1*OE T2 and *tvhydii1*OE T5, we put a 20 μL drop of distilled water and observed its permanence over the mycelium.

### *Tvhydii1* knockout and overexpressing strains mycoparasitism assay

For *tvhydii1* mycoparasitic characterization, *T. virens* Gv29-8, *R. solani* AG2 and AG5 were grown on potato dextrose agar plates. Δ*tvhydii1* T2.1 and Δ*tvhydii1* T2.3 were grown on Vogel minimal medium, *tvhydii1*OE T2 and *tvhydii1*OE T5 were grown on potato dextrose agar plates supplemented with 50 μg/mL of hygromycin. All strains were grown at 28 °C for 72 h. Plugs of mycelia from each *T. virens* strain and both pathogens were place in opposite sides a potato dextrose agar plate for the confrontation assays, and kept at 28 °C in total darkness, for 14 days. Plates were observed daily, and photographs were taken from the front and back of each plate.

### Tomato root colonization assay

*Solanum lycopersicum* seeds were placed in pots containing vermiculite until germination. After 10 days of germination, 1x10^3^ conidia from either *T. virens* Gv29-8, knockout or overexpressing strains, were inoculated in each pot. After 10 days post inoculation, roots were washed with distilled water several times to eliminate any fungus or vermiculite that could be attached to the root surface. Roots were stored at −80 °C until further analysis. Three replicates were performed.

### DNA extraction

Root DNA was extracted following the protocol reported by Reader & Broda [99], using a urea buffer (urea 42%, NaCl 0.3 M, EDTA 0.02 M, Tris-HCl pH 8 0.05 M).

### Gene amplification assay by qPCR

qPCR amplification assays were carried out with 150 ng of root DNA as template. *Sm1* gene from *T. virens* was used to identify the fungal DNA, and we used *expressed* gene [62] to distinguish de plant DNA. The *expressed* gene primers amplify a DNA fragment suitable for real time PCR (300pb).

### Primer design

Primer sequences were design using the QuantPrime [100] and Primer Quest [101] online tools, and the SnapGene program (GSL Biotech, LLC). Primers for qRT-PCR were designed to produce amplicons around 200 bp. For the list of the primers used in this study, see Additional file 5.

### Statistical analysis

A two-way ANOVA and a Bonferroni *post hoc* test were carried out to compare the data obtained from the RT-PCR and qRT-PCR assays, using the GraphPad Prism 4 software (GraphPad Software, Inc.).

## Additional files

Additional file 1: Methodology to obtain possible effector coding genes from *Trichoderma* spp. Complete description of the pipeline we followed to obtain a list of possible effector coding genes from *T. virens, T. atroviride* and *T. reesei*. (DOCX 39 kb)
Additional file 2: Datasets. Lists of proteins that constitute the negative, positive and evaluation datasets used for the comparison of the programs that predict extracellular proteins. (XLSX 20 kb)
Additional file 3: Program performance evaluation. Program performance evaluation of different algorithms tested for their ability to differentiate extracellular proteins from non-extracellular proteins. a) The ROC space and plot of the eleven algorithms evaluated. b) Matthew’s correlation coefficient (MCC) from the eleven algorithms evaluated. Receiver operating characteristic (ROC) graph depicts relative trade-offs between true positive (benefits) and false positive (costs) as x and y axes respectively. The best possible prediction method would yield a point in the upper left corner of the ROC space, representing 100% sensitivity and 100% specificity. The asterisk (*) highlights the algorithm with the best evaluation. (PPTX 160 kb)
Additional file 4: Catalogue of *Trichoderma* effector candidates. Complete list of the possible effector coding genes from *T. virens, T. atroviride* and *T. reesei* that we obtained from our pipeline. (XLSX 21 kb)
Additional file 5: Primers. Complete list of primers used in this study. (XLSX 14 kb)

## Abbreviations

BC: Before contact
C: Contact
CFEM: Common in fungal extracellular membranes
LysM: Lysine motif
MCC: Matthew’s correlation coefficient
OG: Overgrowth
RXLR: Arginine – any amino acid – leucine – arginine
