# Supplementary material for: Identification of effector-like proteins in Trichoderma spp. and role of a hydrophobin in the plant-fungus interaction and mycoparasitism
Source: BMC Genet. 2017 Feb 15;18:16. doi: 10.1186/s12863-017-0481-y (PMC5310080; doi:10.1186/s12863-017-0481-y)
Supplement: Additional file 1: — Methodology to obtain possible effector coding genes from Trichoderma spp. Complete description of the pipeline we followed to obtain a list of possible effector coding genes from T. virens, T. atroviride and T. reesei. (DOCX 39 kb) [file 12863_2017_481_MOESM1_ESM.docx]

**Methodology to obtain possible effector coding genes from *Trichoderma* spp.**

**1. Genome datasets**

Predicted extracellular proteins of ascomycete fungi were downloaded from Fungal Secretome Knowledge Base -FunSecKB- [1]. Predicted proteome datasets for *T. virens, T.reesei* and *T. atroviride* were downloaded from the JGI website [2] and from the National Center for Biotechnology Information –NCBI [3].

**2. Evaluation of programs for the prediction of extracellular proteins**

*2.1. Protein datasets*

To build the test datasets we required only experimentally proven extracellular proteins. We used two main strategies, first we used annotations provided within entries of the UniProtKB/Swiss-Prot database to integrate our negative datasets and second, we performed a text-mining in literature to look for direct experimental evidence of our positive extracellular protein set.

We used regular expressions for the purpose of database searches. In both cases proteins marked as 'fragment' and those whose annotations are reported as 'possible', 'probable', and 'by similarity' were excluded from the correspondent dataset. Five datasets were therefore collected: four negative and one positive dataset (Additional file 2). The description of the datasets is next.

1) A dataset with cytoplasmic localization was built with the keyword (KW) “Cytoplasmic”. We complemented this with the KW “Confidence: experimental” and the KW “evidence at protein level". We further restrict the search with the KW “taxonomy: Dikarya”. Our final sentence for search was: cytoplasmic AND annotation: (type: location "cytoplasm" confidence: experimental) AND existence: "evidence at protein level" AND taxonomy: Dikarya). All the sequences retrieved by this search were downloaded, filtered using PERL scripts for those sequences with more than one sub-cellular localization and finally revised manually for possible inconsistencies in the annotation. These sequences were considered as the cytoplasmic negative dataset.

2) A dataset with mitochondrial localization was constructed with the keyword (KW) “Mithocondrion”. The subsequent steps were similar for those with cytoplasmic localization. Our final sentence for this search was: Mitochondrion AND existence: “evidence at protein level” AND taxonomy: “Dikarya”. All sequences retrieved by this search were downloaded, filtered using PERL scripts and finally revised manually for possible inconsistencies in the annotation. All these sequences were considered as the mitochondrial negative dataset.

3) A dataset with membrane localization was checked with the KW “membrane”. The subsequently steps were similar for those with cytoplasmic localization. Our final sentence for this search was: membrane AND existence: "evidence at protein level" AND taxonomy: dikarya AND annotation: (type: location "Cell membrane"). All the remaining sequences were considering as the membrane negative dataset.

4) To test if the programs can distinguish between classical and non-classical extracellular proteins, effector proteins secreted by non-classical secretory pathways with evidence at protein level reported in literature were identified typing searches in Pubmed [4]. The search was restricted to fungi. In all cases the mining was manually revised and the correspondent sequences downloaded using the protein database from the NCBI. All the selected sequences were considered as the NCSP negative dataset.

*2.1.1 Positive datasets*

To identify the positive dataset the reasoning was simple, all effectors should be extracellular proteins, but not all extracellular proteins are effectors. In addition, we wanted to make sure that all effectors had direct experimental evidence, for this reason we developed a pipeline to mine papers that report effectors in fungal genomes. First, we performed a simple search in Pubmed [4] with the KW “effector protein” limited to reviews. This search allowed us to download a couple of articles with general information about effector proteins [5,6]. Then the PDF papers were transformed to plain text and used the on-line software Wordle [7] to create a word cloud, using this visual representation we mined the most used words in these articles. These new KWs mined were used again to perform searches in Pubmed with Boolean operators. The searches were restricted to articles under complete text section and submitted between 1991 (this is the first report of fungal effector proteins) and August 2011. All the retrieved articles were downloaded and transformed into plain text. The new archives were filtered locally with textpresso software [8] in order to look for papers were KWs appeared in co-occurrence. Finally, the remaining articles were manually reviewed and the correspondent effector amino acid sequence downloaded from the protein database of NCBI. These sequences integrated the positive effector dataset.

*2.2. Program performance evaluation*

Phobius [9], and ProtCompV9 [10] web server predictors were interrogated to test our datasets, while WolfPsort [11], SignalPv3 NN and SignalPv3-HMM [12] as well as SignalPv4 were run locally with the latest free available distribution. In order to assess each prediction program performance, several statistical values were calculated. In the following formulas we indicate with TP and TN the number of True Positive and True Negative predictions, respectively, and with FP and FN the number of False Positive and False Negative predictions, respectively.

The recall (Rc), or true positive rate, was calculated as the number of proteins correctly predicted as extracellular over the total number of positive examples, Rc = TP / (TP + FN). The precision (Pr) corresponds to the number of proteins correctly predicted as extracellular over the total number of protein predicted as extracellular, Pr = TP / (TP + FP). The false positive rate (Fpr) corresponds to the number of protein predicted as extracellular but annotated as negative, Fpr = FP / (FP + TN). The accuracy (Acc) is the proportion of all true predictions on positive and negative examples and is given by Acc = (TP + TN) / (TP + TN + FN + FP).

Finally, Mathews’ correlation coefficient (MCC) is given by MCC = (TP*TN)-TN*FN)/√(TP+FN) (TP+FP) (TN+FP) (TN +FN). It varies between -1 and +1. A value of +1 indicating there is a perfect agreement between observations and predictions; a value of 0 indicating that predictions are not better as they were randomly generated; a value of –1 indicating that predictions are in total disagreement with what is observed.

**3. Building and automating own pipeline to identify effectors at genomic-scale**

*3.1. Automating pipeline to identifying extracellular proteins*

In order to identify possible extracellular proteins in *Trichoderma* species at genomic level two homemade PERL script were developed. The first script performed the signal peptide searches using SignalP (which was the best evaluated algorithm, see above), which calls the second script -Secretor-, which parses all extracellular proteins. These two steps are performed for every genome analyzed. The scripts are available upon request.

*3.2. Clustering of predicted extracellular proteins*

Sets of predicted extracellular proteins from each *Trichoderma* genome were clustered using ‘‘all against all’’ BLASTp [13] followed by TribeMCL [14].

*3.3. Composition analysis and enrichment of the predicted extracellular proteins in* Trichoderma

Functional characterization of extracellular proteins was based on GO annotation and carried out by means of the universal platform Blast2GO [15] version 2.6.0, with default parameters. For each set of extracellular proteins, GO term enrichment analysis were carried out through the Blast2GO suite [16], applying Fisher’s exact test as implemented in GOSSIP [17] GO terms with a False Discovery Rate (FDR) q-value ≤ 0.05 were considered significantly enriched in each comparison.

*3.4. Looking for effector features in extracellular proteins*

3.4.1. Homologs of effectors in the *Trichoderma* genomes

The effector positive dataset was used as query in order to identify homologs to known effectors in the *Trichoderma* genomes. BLAST searches were conducted with an e-value cutoff of 10E-2. All sequences with significant matches were parsed using PERL scripts. We selected parsed hits if, 1) the percentage of identity is equal or more than 30%; 2) the alignment length was equal or longer than 50 aa; 3) the coverage of the alignment was equal or over than 30%. Finally selected hits were revised manually for inconsistencies.

3.4.2. Identification of Pfam domain in *Trichoderma* genomes

The Pfam-A library from release 25.0 of the Pfam database [18] was downloaded from the Pfam website [19]. This library contains 14831 protein models constructed from manually curated multiple alignments. This library was used to analyze the sequences of the effector positive dataset as well as the predicted extracellular proteins of the *Trichoderma* genomes to identify the Pfam domain that each protein contains. The analysis was performed using the ‘‘hoz.pl’’ PERL script (available upon request), where similarity is detected using the HMMER3 [20] suite of programs. Default thresholds were used, which are hand-curated for every family and designed to minimize false positives. We then identified all Trichoderma extracellular proteins with similar domain structure to our validated dataset using Perl scripts.

3.4.3. Identification of host localization signals in *Trichoderma* genomes

We searched each protein for effector motifs validated experimentally as ‘RxLR’ [21], RxFLAK [22], W/Y/F-XC [23], and possible variants [RKH]x[LYMFYW][RKH] [24] between amino acids 15 to 75 using Perl scripts. Nuclear localization signals were predicted with Predicts [25].

3.4.4. De novo motif analysis in extracellular proteins

De novo protein motif search was performed on extracellular proteins in tribes using MEME [26]. The program was set to report the 25 most robust motifs of 4 to 10 amino-acids, occurring zero or once per sequence, among the extracellular proteins. The motifs were classified based on the dispersion of their position along the protein sequence, and the number of tribes in which they were found. Motifs showing reduced dispersion (interquartile range for motif position, 10 amino acids) and found in at least three proteins were considered as conserved and reported.

3.4.5. Identification of tandem repeats in extracellular proteins in *Trichoderma* genomes

The presence of tandem repeats (TR's) in the extracellular dataset of *T. atroviride, T. virens* and *T. reseei* were determined using the T-reks software locally [27] with the default parameters. Homemade PERL scripts were developed to parse and analyze the data.

3.4.6. Identification of SSCRP's in *Trichoderma* genomes

Small Secreted Cysteine Rich Proteins (SSCRP) were identified using a cutoff of 300 amino acid length and an enrichment of cysteins equal to or larger than 3% (defined as the number of cysteins divided by amino acid length and multiplied by 100). All the sequences selected were analyzed for all the possible disulphide bridges using DIpro 2.0 -Protein Disulfide Bond Prediction- [28]. Sequences with cutoffs for enrichment and amino acids length as well as one or more disulphide bridges prediction were considered SSCRP.

3.4.7. Ranking and clustering effector protein predictions

A numerical value was associated to each of the eight-effector properties analyzed from the complete sets of *Trichoderma* proteomes. Then a hierarchical clustering analysis was conducted using MEV4 [29]. The scores associated to each property for proteins in tribes were considered as ‘intensity’ values. The hierarchical tree was optimized using 1000 bootstrap runs with Pearson correlation coefficient as distance value, and average linkage between groups. The priority of effector properties used for clustering was set manually.

**References**

1. Fungal Secretome KnowledgeBase (FunSecKB). http://bioinformatics.ysu.edu/secretomes/fungi.php Accessed 2011 Jun 10.

2. JGI Fungi Portal - Home. http://genome.jgi.doe.gov/programs/fungi/index.jsf Accessed 2011 Jun 10.

3. Home - Genome - NCBI. https://www.ncbi.nlm.nih.gov/genome Accessed 2011 Jun 12.

4. Home - PubMed - NCBI. https://www.ncbi.nlm.nih.gov/pubmed Accessed 2011 Jul 6.

5. Stergiopoulos I, de Wit PJGM. Fungal effector proteins. Annu. Rev. Phytopathol. 2009;47:233–63.

6. De Wit PJGM, Mehrabi R, Van den Burg HA, Stergiopoulos I. Fungal effector proteins: past, present and future. Mol. Plant Pathol. 2009;10:735–47.

7. Wordle - Beautiful Word Clouds. http://www.wordle.net/ Accessed 2011 Jul 7.

8. Textpresso Home. http://www.textpresso.org/ Accessed 2011 Jul 8.

9. Phobius. http://phobius.sbc.su.se/ Accessed 2011 Jul 24.

10. ProtComp - Predict the sub-cellular localization for Animal/Fungi proteins. http://www.softberry.com/berry.phtml?topic=protcompan&group=programs&subgroup=proloc Accessed 2011 Jul 8.

11. WoLF PSORT: Advanced Protein Subcellular Localization Prediction Tool - GenScript. http://www.genscript.com/wolf-psort.html Accessed 2011 Jul 9.

12. SignalP 3.0 Server. http://www.cbs.dtu.dk/services/SignalP-3.0/ Accessed 2011 Jul 9.

13. BLAST: Basic Local Alignment Search Tool. https://blast.ncbi.nlm.nih.gov/Blast.cgi? Accessed 2011 Jul 22.

14. Saunders DGO, Win J, Cano LM, Szabo LJ, Kamoun S, Raffaele S. Using Hierarchical Clustering of Secreted Protein Families to Classify and Rank Candidate Effectors of Rust Fungi. Stajich JE, editor. PLoS One. 2012;7:e29847.

15. Blast2GO - Functional Annotation and Genomics. https://www.blast2go.com/ Accessed 2011 Aug 1.

16. Götz S, García-Gómez JM, Terol J, Williams TD, Nagaraj SH, Nueda MJ, et al. High-throughput functional annotation and data mining with the Blast2GO suite. Nucleic Acids Res. 2008;36:3420–35.

17. Conesa A, Götz S, García-Gómez JM, Terol J, Talón M, Robles M. Blast2GO: a universal tool for annotation, visualization and analysis in functional genomics research. Bioinformatics. 2005;21:3674–6.

18. Bateman A, Birney E, Durbin R, Eddy SR, Howe KL, Sonnhammer EL. The Pfam protein families database. Nucleic Acids Res. 2000;28:263–6.

19. Pfam @ Wellcome Trust Sanger Institute. https://pfam.sanger.ac.uk/ Accessed 2011 Aug 25.

20. HMMER. http://hmmer.org/ Accessed 2011 Aug 30.

21. Dou D, Kale SD, Wang X, Jiang RHY, Bruce NA, Arredondo FD, et al. RXLR-Mediated Entry of Phytophthora sojae Effector Avr1b into Soybean Cells Does Not Require Pathogen-Encoded Machinery. Plant Cell. 2008;20:1930–47.

22. Liu T, Ye W, Ru Y, Yang X, Gu B, Tao K, et al. Two host cytoplasmic effectors are required for pathogenesis of Phytophthora sojae by suppression of host defenses. Plant Physiol. 2011;155:490–501.

23. Godfrey D, Böhlenius H, Pedersen C, Zhang Z, Emmersen J, Thordal-Christensen H. Powdery mildew fungal effector candidates share N-terminal Y/F/WxC-motif. BMC Genomics. 2010;11:317.

24. Kale SD, Tyler BM. Entry of oomycete and fungal effectors into plant and animal host cells. Cell. Microbiol. 2011;13:1839–48.

25. PredictNLS - Rost Lab Open. https://rostlab.org/owiki/index.php/PredictNLS Accessed 2011 Oct 4.

26. Introduction - MEME Suite. http://meme-suite.org/ Accessed 2011 Sep 9.

27. BiSMM - Structural Bioinformatics and Molecular Modeling. http://bioinfo.montp.cnrs.fr/?r=t-reks/ Accessed 2011 Sep 25.

28. Dipro server. http://download.igb.uci.edu/bridge.html Accessed 2011 Sep 30.

29. MeV download | SourceForge.net. https://sourceforge.net/projects/mev-tm4/ Accessed 2011 Oct 5.
