# Supplementary figures and images for: Identification of effector-like proteins in Trichoderma spp. and role of a hydrophobin in the plant-fungus interaction and mycoparasitism
Source: BMC Genet. 2017 Feb 15;18:16. doi: 10.1186/s12863-017-0481-y (PMC5310080; doi:10.1186/s12863-017-0481-y)

## Slide 1
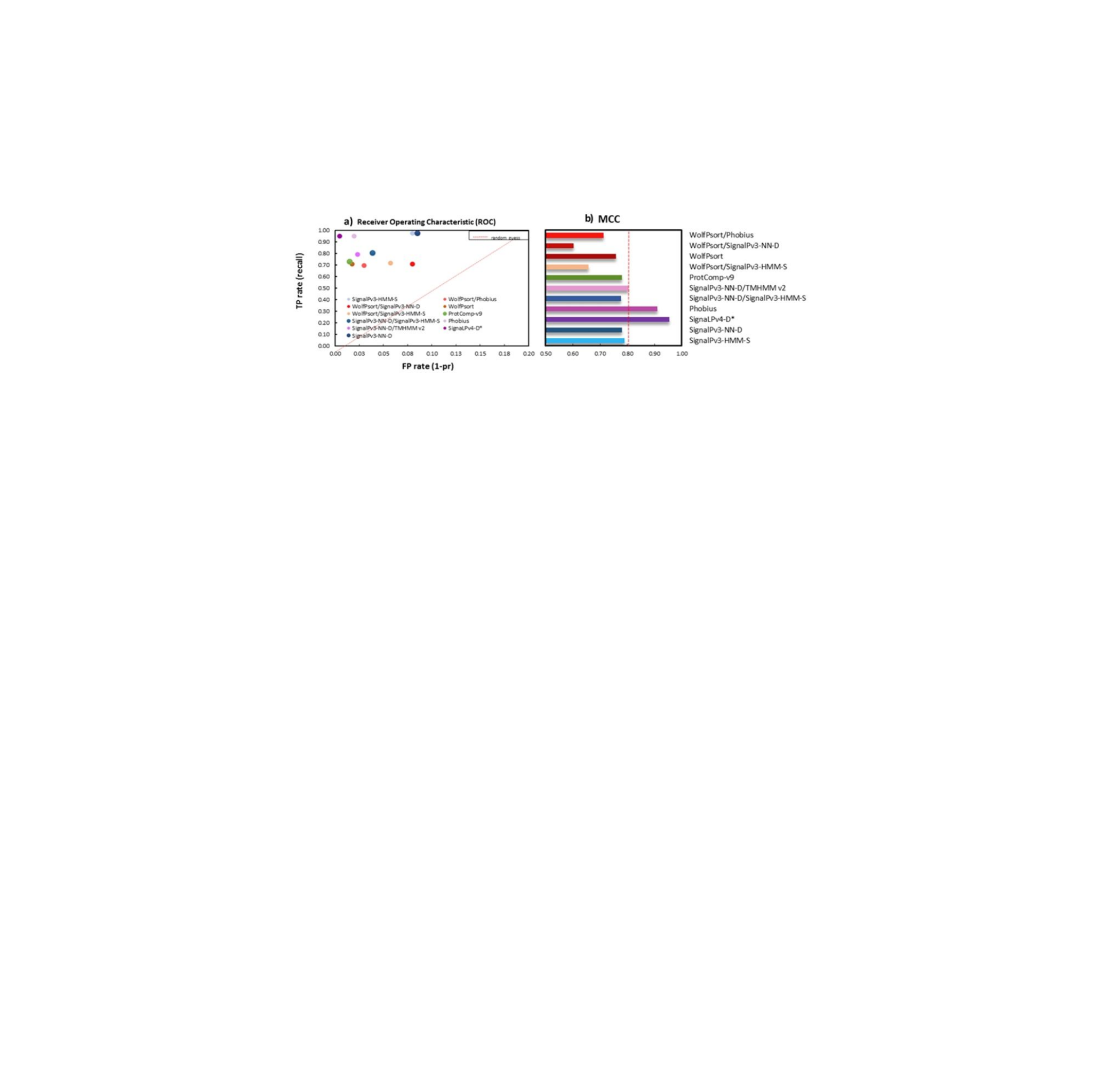

Supplement: Additional file 3: — Program performance evaluation. Program performance evaluation of different algorithms tested for their ability to differentiate extracellular proteins from non-extracellular proteins. a) The ROC space and plot of the eleven algorithms evaluated. b) Matthew’s correlation coefficient (MCC) from the eleven algorithms evaluated. Receiver operating characteristic (ROC) graph depicts relative trade-offs between true positive (benefits) and false positive (costs) as x and y axes respectively. The best possible prediction method would yield a point in the upper left corner of the ROC space, representing 100% sensitivity and 100% specificity. The asterisk (*) highlights the algorithm with the best evaluation. (PPTX 160 kb) [file 12863_2017_481_MOESM3_ESM.pptx]
